# The relative risk of infection in people with multiple sclerosis using disease-modifying treatment: a systematic review of observational studies

**DOI:** 10.1007/s10072-025-08018-9

**Published:** 2025-02-08

**Authors:** M. W. Y. Leung, E. M. W. Van de Garde, B. M. J. Uitdehaag, O. H. Klungel, M. T. Bazelier

**Affiliations:** 1 Division Pharmacoepidemiology & Clinical Pharmacology, Department of Pharmaceutical Sciences, Faculty of Science, Utrecht Institute for Pharmaceutical Sciences (UIPS), Utrecht University, Utrecht, The Netherlands; 2 Department of Neurology, MS Center Amsterdam, Amsterdam Neuroscience, Amsterdam University Medical Center, Amsterdam, The Netherlands

**Keywords:** Multiple sclerosis, Infections, Systematic review, Observational research, Disease-modifying treatment

## Abstract

**Background:**

Some disease-modifying treatments (DMTs) for multiple sclerosis (MS) increase the risk of infection, but it remains unknown how the risk compares between trials and observational studies.

**Objective:**

To assess the current state of observational research on the risk of infection in people with MS and using DMTs.

**Methods:**

PubMed and Embase were searched for observational studies published on or before 4 April 2023 describing infection in people with MS, with a comparison of at least 1 DMT to no DMT or another DMT. We examined which DMT contrasts and types of infection were studied and how often; and compared observational results of the most frequently studied DMT to trial data from a network meta-analysis.

**Results:**

Out of 5373 search records 22 papers were eligible, of which 5 reported relative risks (RRs). In total, 9 DMTs were studied. Out of 45 possible contrasts, 9 were not studied, and 19 once. The most assessed specific type of infection was neurological (*n* = 11/22 studies). Natalizumab was the most studied DMT contrasting 7 other DMTs or no DMT, with 12 RRs reported. Point estimates of the RRs (compared to no DMT) for respiratory and urinary tract infections were in opposite direction compared to trial data.

**Conclusion:**

Observational study data on the risk of infection in people with MS on DMT are sparse. The growing availability of real-world data on MS and DMT use provides an opportunity to study specific infections on DMT, which is particularly valuable to populations underrepresented in trials.

## Introduction

Multiple sclerosis (MS) is a chronic disease in which inflammation occurs in the central nervous system. The inflammation can be reduced by disease-modifying treatments (DMTs) for MS. Sixteen DMTs have been approved by the European Medicines Agency to treat relapsing-remitting MS so far. The main factors in treatment decisions for MS include patient preferences and prognostication of the benefit-risk ratio of the treatment [1]. Known risks include infections, but risk stratification for infections remains challenging [2] as infectious risks over the longer term and for rarer events might not occur in trials and the amount of real-world experience differs between the DMTs [3].

The risk of infection during DMT use for MS has been described in several reviews. Some reviews focused on findings from trials only [4, 5], whereas others included observational studies as well but with a particular selection of infection outcomes [6]. Other reviews in turn have compared safety outcomes in general between trials and real-world studies [7, 8], but did not further specify infectious episodes. It thus remains unknown how the risk of infection on DMT for MS compares between trials and observational studies, which is relevant because real-world DMT users are not identical to DMT trial participants [9–11] and may be treated differently.

Therefore, the aim of this systematic review was to assess the current state of observational research on the risk of infection in people with MS (pwMS) and using disease-modifying treatment DMT.

## Methods

### Eligibility criteria

PubMed and Embase were searched for observational studies published on or before 4 April 2023 describing the effect of DMT use on the occurrence of adverse events in pwMS, in which at least two DMTs were compared to each other or at least one DMT to no DMT. Exclusion criteria for study characteristics were: reviews, meta-analyses, letters, commentaries, case reports, case series, randomized studies, clinical trials including studies using trial data, guidelines, expert consensus reports, simulation studies, modelling studies, in vitro studies, and protocols; population-related exclusion criteria were: pediatric population of DMT users, non-human studies, and users of DMT for an indication other than MS; intervention-related exclusion criteria were: treatments not approved by the European Medicines Agency (EMA) for the treatment of MS and infection prevention strategies studied as intervention; and outcome-related exclusion criteria were: studies not reporting on the occurrence of infection, descriptive but no quantitative results, and Sars-CoV-2 as primary infection outcome.

### Search strategy

The search strategy was aimed at studies of a population of pwMS, all EMA-approved DMTs for the treatment of MS, with no DMT as comparator or DMT as active control, and infection as outcome. The full search strategy is shown in the Supplementary file, Table 1.

### Selection process

Rayyan [12] was used to remove duplicate articles, filter on English language, and manually screen articles for eligibility. One reviewer (ML) manually screened all titles and abstracts of the search results from PubMed for eligibility. If the eligibility was uncertain based on title and abstract, the full text was screened on eligibility criteria. If the main text referred to reporting of infections in supplemental data, the supplemental data was screened. A second reviewer (MM) screened a random sample of 200 of the PubMed search results in an identical manner. After the manual screening of the Pubmed search results, ASReview [13] was used in the selection process: the unlabelled dataset of search results from Embase and the labelled dataset of search results from PubMed were uploaded to ASReview, whose algorithm ranked the unlabelled search results from Embase by order of relevance for inclusion in the study. The default settings were used: feature extraction technique TF-IDF, Naïve Bayes classifier, maximum query strategy, and dynamic resampling (double) as balance strategy. The stop criterion was 150 irrelevant records in a row.

### Data collection process

One reviewer collected data from selected papers. The outcome data were infection-related findings. Where available, relative outcome measures of infection with at least one contrast in DMT were collected. If not, the incidence rate of infection was collected, or the number of events of infection if the incidence rate was not reported. The other variables for which data were collected were: aim, study design, data source, exposure, study size, age, sex, and follow-up duration.

### Study risk of bias assessment

The risk of bias in each study with relative risk of infection on different DMTs was assessed using the Risk Of Bias In Non-randomised Studies– of Interventions (ROBINS-I) tool [14]. ML assessed the risk of bias for all studies; MB and OK assessed the risk for two studies, and EG for one study, independently of each other and ML. The discrepancy between assessments was resolved through discussion until a consensus was reached. The results were visualized using the robvis [15] tool.

### Synthesis methods

#### Distribution of DMT contrasts

For each DMT contrast in the observational studies reporting the occurrence of infections on DMT, the number of studies reporting the contrast was summed and visualized in a matrix. If a study grouped DMTs into a single exposure category, this counted as a contrast between each of the DMTs in the one category and each of the DMTs in the other category. Each study could contribute only once to each contrast.

#### Distribution of infection outcomes

The infection outcomes in the observational studies were categorized as any/unspecified, gastrointestinal, genital, herpes viral, neurological, respiratory, sepsis, skin and subcutaneous, or urinary. For each infection type, the number of studies reporting it was summed. If a study reported multiple infection types, it contributed to each of the types.

#### Infection risk on natalizumab: observational data versus network meta-analysis of trial findings

As one example of the comparison between observational data and trial data, results of the infection risk on natalizumab compared to another DMT from observational data were visualized in a matrix alongside the findings from a recent network meta-analysis [5] of trial findings. The distinctions of interest were the direction of the effect, whether the observational studies reported a relative outcome measure, and whether the result was statistically significant. This was studied for each infection type found in an observational study reporting the occurrence of infection on natalizumab with at least one contrast to another single DMT or no DMT. Discrepant results in the network meta-analysis between outcomes of the same infection type or between dosages were plotted separately.

## Results

### Study selection

**Fig. 1:**
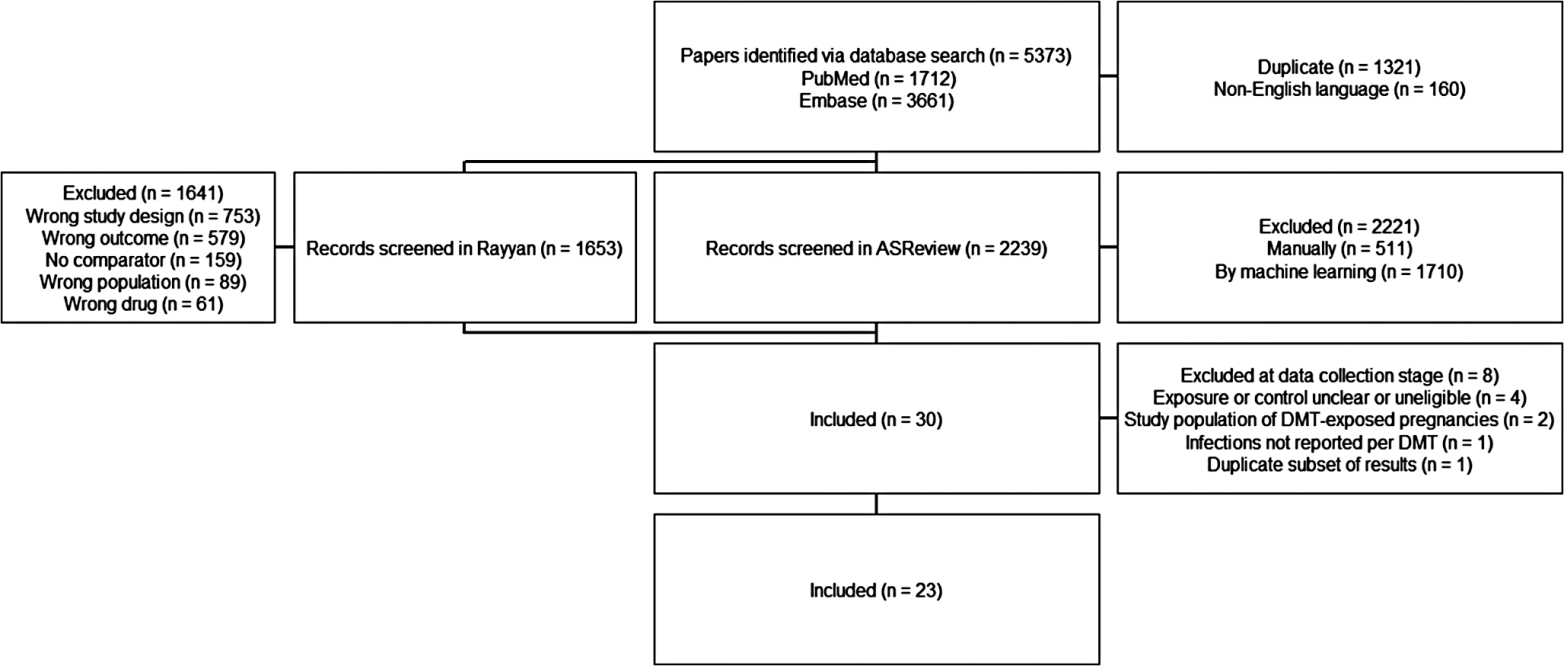
Flowchart of study inclusion

The search strategy retrieved 1712 records from PubMed and 3661 from Embase, resulting in 5373 in total. 3892 records remained after filtering on English language and deduplication. 1653 of these were from PubMed and manually screened on title and abstract, and 12 were included. The partially labelled dataset of 3892 records was subsequently uploaded to ASReview, where the stopping rule was reached after 529 screened records, of which 18 labeled relevant. 30 papers out of 3892 search results were thus included in the selection process. However, 8 studies were later excluded: two papers included unclear control exposures, one compared two interferon beta treatments to each other, one did not describe which treatments were included in ‘any DMT’, two reported on DMT-exposed pregnancies, one did not report infection events per DMT, and one reported a subset of results that were reported in another included study. 22 papers were ultimately included in this review (Fig. 1), of which five reported a relative outcome measure of infection, two reported incidence rates and fifteen reported numbers of events.

### Study characteristics

The aim, study design, data source, exposure, study size, age, sex, and follow-up duration of the included studies are shown in Table 1.

**Table 1:** Characteristics of the included papers

Study	Study aim	Study design	Data source	Exposure	Study size	Age	Sex	Follow-up time
**Studies reporting relative risk of infection on different DMTs**								
**De Jong et al.** [16]	To examine the association between IFN-beta and potential adverse events using population-based health administrative data in British Columbia, Canada	Retrospective cohort study with nested case-control analyses of potential adverse events with > = 30 cases regardless of treatment exposure	British Columbia Multiple Sclerosis (BCMS) database linked to PharmaNet (filled drug prescriptions), Medical Serice Plan Payment information (physician visit dates with diagnoses coded using International Classification of Diseases (ICD)-9/10), Discharge Abstract Database (hospital discharge diagnoses, coded using ICD-9/10), Census Geodata (aggregated data on neighbourhood socioeconomic status), Vital Statistics (death dates), BC Registration and Premium Billing File (registration status in mandatory provincial health care plan residency in BC, age, sex)	IFN-beta	**n = 2485 people with RRMS, cases (n)/controls (n)** **Infections**: 863/14,216; **Upper respiratory infection (URI)**: 1037/16,173; **Otitis media**: 259/4669; **Pneumonia**: 213/3588.	**Mean (SD) in years, cases/controlS** **Infections**: 41.8 (10.4)/41.8 (9.4); **URI**: 41.2 (9.6)/41.3 (8.7); **Otitis media**: 40.0 (9.4)/40.2 (8.5); **Pneumonia**: 44.5 (10.8)/44.1 (9.6)	**Women, n (%) cases/controlS** **Infections**: 703 (81.5)/703 (81.5); **URI**: 836 (80.6)/836 (80.6); **Otitis media**: 210 (81.1)/210 (81.1); **Pneumonia**: 161 (75.6)/161 (75.6)	**Mean (SD) in years, cases/control** **s** **Infections**: 3.6 (2.9)/3.6 (2.9); **URI**: 2.7 (2.5)/2.7 (2.5); **Otitis media**: 4.2 (3.2)/4.2 (3.3); **Pneumonia**: 5.2 (3.2)/5.2 (3.2)
**Luna et al.** [17]	To examine the risk of serious infections associated with DMT for MS	Retrospective cohort study	Swedish MS register	IFN-beta or GA, FTY, NAT, (RTX)	**6421 pwMS with 8600 treatment episodes. Treatment initiations (n)** **IFN-beta and GA**: 2217; **FTY**: 1535; **NAT**: 1588; **RTX**: 3260.	**Mean (SD), years** **Overall**: 39.0 (10.7); **IFN-beta and GA**: 40.1 (11.3); **FTY**: 38.8 (9.6); **NAT**: 35.0 (10.1); **RTX**: 40.4 (10.6).	**Women, n (%)** **Overall**: 6186 (71.9); **IFN-beta and GA**: 1631 (73.6); **FTY**: 1045 (68.1); **NAT**: 1152 (72.5); **RTX**: 2358 (72.3).	**Total (mean), years** **IFN-beta and GA**: 4688 (2.1); **FTY**: 4129 (2.7); **NAT**: 3969 (2.5); **RTX**: 6533 (2.0).
**Nicholas et al.** [18]	To compare infection-related healthcare resource utilization and healthcare costs between pwMS treated with DMF or OCR	Retrospective cohort study	Optum Clinformatics Data Mart database (United States of America), patient-level data using administrative, pharmacy, physician, and facility claims primarily from a private insurance group)	DMF, OCR	**Users, n, propensity score matching populations** **OCR**: 1094; **DMF**: 1094.	**Mean (SD) in years, propensity score matching populations** **OCR**: 49.3 (12.6); **DMF**: 50.4 (12.8).	**Women, n (%), propensity score matching populations** **OCR**: 818 (75); **DMF**: 827 (76).	**Mean (SD), days, unadjusted populations (n = 3170 OCR users**, *n* = 1429 DMF users) **DMF**: 296 (244); **OCR**: 297 (243).
**Wijnands et al.** [19]	To assess the association between the DMTs approved for use in MS and risk of infections in a population-based setting	Retrospective cohort study	Population Data British Columbia: Medical Service Plan Billing Information and Discharge Abstract Database (physician claims and hospital admissions); PharmaNet (drug prescriptions filled); Census Geodata (socioeconomic status); Registration and Billing files (residency); and Vital Statistics Deaths (death dates)	TERI, ALE, IFN-beta, GA, NAT, FTY, DMF	**n = 6793 pwMS;** 1716 (25.3%) ever exposed to any DMT	**Mean (SD), years**: 45.4 (13.3)	**Women, n (%)**: 4999 (73.6%)	**Median [IQR], years**: 8.5 [4.6–12.7]
**Zappulo et al.** [20]	To investigate the incidence of infections and associated prognostic factors during the first year of treatment in people receiving anti-CD20 (OCR or RTX) or anti-CD52 monoclonal antibodies (ALE)	Retrospective cohort study	Paper charts and electronic medical records from the Centre of Neurodegenerative Diseases of Naples at the University of Naples Federico II	ALE, OCR (or RTX)	**Users, n** **Overall:** **163;** **Anti-CD20**: 96; **Anti-CD52**: 67.	**Mean (SD), years** **Overall**: 44.5 (11.4); **Anti-CD20**: 48.4 (10.3); **Anti-CD52**: 38.9 (10.5).	**Women, n (%)** **Overall**: 100 (61); **Anti-CD20**: 50 (52); **Anti-CD52**: 50 (75).	**Median [IQR], days** **Overall**: 226 [96–365]; **Anti-CD20**: 133 [64–231]; **Anti-CD52**: 365 [345–365].
**Studies reporting rates of infection on different DMTs**								
**Alping et al.** [21]	To assess safety outcomes for the induction therapies ALE and AHSCT compared to noninduction DMTs	Retrospective cohort study	Swedish MS register linked to cause of death register, patient register (visits with associated diagnosis codes to inpatient and specialized outpatient care), prescribed drug register (prescription drugs collected at pharmacies), cancer register, demographic registers, and registers with data on sick leave and disability pension	ALE, FTY, NAT, DMF, (AHSCT, RTX)	**Users, n** **ALE**: 132; **Reference DMT, matched to ALE and AHSCT groups**: 2486 (36% natalizumab, 29% DMF, 22% rituximab, 13% fingolimod).	**Mean (SD), years** **ALE**: 35.0 (7.8); **Reference DMT, matched to ALE and AHSCT groups**: 33.9 (7.5).	**Women, n (%)** **ALE**: 79 (59.8); **Reference DMT, matched to ALE and AHSCT groups**: 1627 (65.4).	**Mean, years** **ALE**: 3.5; **Reference DMT, matched to ALE and AHSCT groups**: 4.3 or 3.6 (depending on outcome)
**Simbrich et al.** [22]	To describe drug-use patterns in pwMS using DMTs and estimate the incidence of SAEs of treatment	Retrospective cohort study	German Pharmacoepidemiological Research Database (claims data from statutory health insurance providers in Germany)	GA, IFN-beta, FTY, NAT	**Users per outcome cohort, n: overall/PML/any infectious disease** **IFN-beta**: 6372/5124/5071; **GA**: 4088/3467/3429; **NAT**: 442/324/317; **FTY**: 208/130/128.	**Mean (SD), years** **IFN-beta**: 38.4 (10.1); **GA**: 38.9 (9.4); **NAT**: 36.3 (9.5); **FTY**: 38.9 (10.2).	**Women, %** **IFN-beta**: 68.2; **GA**: 70.9; **NAT**: 66.3; **FTY**: 74.5.	**Total per outcome cohort, years: PML/any infectious disease** **IFN-beta**: 5535.4/5498.0; **GA**: 5916.0/5857.0; **NAT**: 534.0/519.2; **FTY**: 113.7/111.0.
**Studies reporting number of infections on different DMTs**								
**Baharnoori et al.** [23]	To identify predictors of hematological abnormalities in pwMS treated with DMF or FTY, and study the impact of treatment switch on lymphocyte and leukocyte count	Retrospective cohort study	Nested cohort of pwMS in the Comprehensive Longitudinal Investigation of MS at Brigham and Women’s Hospital (CLIMB) and Partners MS Center prospective study	DMF, FTY	**Users, n** **FTY**: 300; **DMF**: 405.	**Mean (SD), years** **FTY**: 40.8 (10.2); **DMF**: 45.3 (11.3).	**Women, n (%)** **FTY**: 221 (73.6); **DMF**: 295 (72.8).	At least 12 months, up to 5 years
**Boffa et al.** [24]	To assess whether lymphopenia is associated with short-term treatment response and infection rate in a real-life MS population treated with FTY and DMF	Retrospective cohort study	Records from the MS Center of the University of Genoa	FTY, DMF	**Users, n** **FTY**: 137; **DMF**: 75.	**Mean (SD), years** **FTY**: 40.5 (10.3); **DMF**: 41.9 (11.1)	**Women, n (%)** **FTY**: 91 (66.4); **DMF**: 44 (58.6)	By design: 12 months
**Boremalm et al.** [25]	To compare the efficacy, safety, and medication persistence NAT, RTX, and FTY as escalation therapy in RRMS	Retrospective cohort study	Swedish MS registry	FTY, NAT, (RTX)	**Users, n** **Overall**: 241 **NAT**: 105; **RTX**: 48; **FTY**: 88.	**Median [IQR], years**: **NAT**: 34.9 [28.9–42.0]; **RTX**: 39.1 [31.7–46.7]; **FTY**: 37.1 [30.9–44.7].	**Women, n (%)** **NAT**: 79 (75.2); **RTX**: 35 (72.9); **FTY**: 58 (65.9).	**Median [IQR], years** **NAT**: 2.8 [1.9–4.5]; **RTX**: 2.8 [2.1–3.6]; **FTY**: 2.6 [1.7–3.8].
**Bose et al.** [26]	To report the safety and efficacy data of ALE and CLA in a real-world, single-center setting	Retrospective cohort study	Institutional electronic medical records and local MS Clinical iMed database at Ottawa Hospital MS Clinic	ALE, CLA	**Users, n** **ALE**: 46; **CLA**: 65.	**Median [IQR], years** **ALE**: 36.1 [31–42]; **CLA**: 43.8 [37–50].	**Women, n (%)** **ALE**: 38 (82.6); **CLA**: 46 (70.8).	**Median [IQR], years** **ALE**: 3.3 [1.0-10.7]; **CLA**: 3.2 [0.3–12.1].
**D’Amico et al.** [27]	To evaluate the achievement of ‘no evidence of disease activity’ over a 12-month period in a large multicenter population with RRMS treated with delayed-release DMF and TERI using a propensity-score adjustment	Prospective cohort study	Retrospective clinical, radiographic and demographic data up to 12 months to treatment start; routinely collected clinical data at baseline and 12 months after treatment initiation from 9 Italian centers	DMF, TERI	**Users, n, propensity score matched populations** **DMF**: 234; **TERI**: 234.	**Mean (SD), years** **DMF**: 45.7 (9.9); **TERI**: 44.9 (9.2).	**Women, n (%)** **DMF**: 148 (63.3); **TERI**: 151 (64.5).	12-month observational period
**Ferro et al.** [28]	To analyze the results of a systematic collaborative approach between Neurology and Infectious Disease Departments in the management of infectious risk and complications in pwMS treated with DMT	Retrospective cohort study	Electronic medical records from the Centro Hospitalar Universitário de São João MS clinic (in the north of Portugal) and its Immunomodulation and Infectious Risk Out-patient Clinic	DMF, FTY, NAT, (RTX)	**Users, n (%)** **Overall**: 149 **NAT**: 82 (55); **FTY**: 73 (49); **RTX**: 17 (11); **DMF**: 32 (21).	**Median [IQR], years**: 37 [29–46]	**Women, n (%)**: 107 (72)	**Median [IQR], months**: 40 [35]; **range**: 6–82 months
**Frisell et al.** [29]	To provide real-world data on safety and discontinuation rates of FTY and NAT	Retrospective cohort study	Swedish MS registry (clinical data), IMSE data, linked to questionnaire data from Epidemiological Investigation of risk factors for MS and Genes and Environment in MS studies, or separate questionnaire data on lifestyle and sociodemographic variables	FTY, NAT	**Users, n** **NAT**: 640; **FTY**: 876	**Mean (SD), years** **NAT**: 36 (10); **FTY**: 38 (10).	**Men, n (%)**: **NAT**: 151 (24); **FTY**: 281 (32).	**Up to 12 months, users on DMT at 12 months, n (%)** **NAT**: 542 (87); **FTY**: 690 (80).
**Gajofatto et al.** [30]	To compare the outcome of people with RRMS treated with NAT or FTY	Retrospective cohort study	Medical charts from a University Hospital in Italy and Italian Drug Agency monitoring registries of Tysabri and Gilenya (demographic and clinical variables)	FTY, NAT	**Users, n** **Overall**: 87; **NAT: 5**7; **FTY**: 30.	**Mean (SD), years** **Overall**: 38.4 (8.8); **NAT**: 38.0 (9.3); **FTY**: 39.0 (7.8).	**Women, n (%)**: **Overall**: 64 (73.6); **NAT**: 43 (75.4); **FTY**: 21 (70).	**Mean (95% CI), months**: 25 (22–28) months
**Harding et al.** [31]	To analyze long-term outcomes in a population-based cohort according to initial treatment strategy	Retrospective cohort study	Population-based cohort of pwMS in southeast Wales	ALE, DMF, FTY, GA, IFN-beta, NAT, TERI. Categorized as early intensive treatment (EIT) if 1st -line was ALE or NAT and as escalation (ESC) otherwise.	**Users, n** **EIT**: 104; **ESC**: 488.	**Mean (SD), years, EIT/ESC** **At symptom onset**: 29.8 (9.2)/30.2 (9.4); **At first DMT**: 34.0 (9.0)/38.5 (9.7).	**Women, n (%)**: **EIT**: 79 (76); **ESC**: 346 (71)	**Mean (SD), years** **EIT**: 5.8 (3.6); **ESC**: 6.9 (5.3).
**Moreira Ferreira et al.** [32]	To analyze the safety and effectiveness of DMF in pwMS	Retrospective cohort study	Partners Oracle Database (Harvard Multiple Sclerosis Patient Database) and CLIMB database	DMF, GA	**Users, n** **DMF**: 46; **GA**: 42.	**Mean (SD), years / median [IQR], years** **DMF**: 56.4 (10.1) / 56.5 [32.1–79.2] **GA**: 52.3 (9.1) / 53.2 [29.4–68.4]	**Women, n (%)** **DMF**: 31 (67.4); **GA**: 27 (64.3).	At least 12 months, up to 80 months
**Prosperini et al.,** [33]	To evaluate the long-term (10 years) effectiveness of initial treatment with escalation (ESC) versus induction (IND) approach in RRMS patients, using a multicenter, retrospective local MS registry data.	Retrospective cohort study	Ad hoc electronic database developed for the study, data collection from 5 tertiary MS outpatient clinics in Italy	ALE, CLA, DMF, FTY, GA, IFN-beta, NTZ, TERI, (AZA, CYC, MTX, RTX). Categorized as ESC if the first DMT was IFN-beta or GA and as IND if it was MTX or CYC.	**Users, n** **ESC**: 738; **IND**: 75.	**Mean (SD), years** **ESC**: 32.2 (8.4); **IND**: 35.6 (9.3).	**Men, n (%)** **ESC**: 212 (28.7); **IND**: 24 (32.0).	10 years
**Rojas et al.,** [34]	To compare the effectiveness and safety of early high-efficacy (EHE) versus escalation (ES) strategies in pwMS from Argentina in a real-world setting	Retrospective cohort study	Data from medical records transferred to specific case report form, from 7 tertiary MS outpatient clinics in Argentina	ALE, CLA, DMF, FTY, GA, IFN-beta, NAT, OCR, TERI, (MTX, RTX). Categorised as ES if starting therapy was DMF, FTY, GA, IFN-beta, or TERI and as EHE otherwise.	**Users, n (matched cohort)** **ES**: 193; **EHE**: 112.	**Mean (SD), years, ES/EHE** **At study entry**: 38.3 (10)/37.8 (9.2); **At disease onset**: 31.2 (9.7)/31.1 (8.6); **At disease diagnosis**: 32.3 (10)/31.7 (87).	**Women, n (%)** **ES**: 106 (55); **EHE**: 70 (62.5).	Up to 96 months
**Vollmer et al.,** [35]	To compare 2-year effectiveness and discontinuation of NAT versus FTY and DMF in the treatment of MS	Retrospective cohort study	Electronic medical records from the Rocky Mountain Multiple Sclerosis Center at the University of Colorado (RMMSC at CU)	DMF, FTY, NAT	**Users, n** **NAT**: 451; **FTY**: 271; **DMF**: 342.	**Mean (SD), years** **NAT**: 39.8 (12.1); **FTY**: 42.5 (11.4); **DMF**: 45.8 (12.2).	**Women, n (%)** **NAT**: 346 (76.7); **FTY**: 195 (72.0); **DMF**: 238 (69.6).	**Up to 24 months. Users on DMT at 24 months, n (%)** **NAT**: 157 (51.6%); **FTY**: 85 (47.8%); **DMF**: 20 (11.0%).
**Vollmer et al., ** [36]	To assess real-world discontinuation, effectiveness, and switching practices of DMF and FTY over 36 months along with disease activity after switching DMT	Retrospective cohort study	Electronic medical records from the Cleveland Clinic Mellen Center and RMMSC at CU	DMF, FTY	**Users, n** **DMF**: 737; **FTY**: 535.	**Mean (SD), years** **DMF**: 46.4 (11.6); **FTY**: 43.3 (10.4).	**Women, n (%)** **DMF**: 516 (70.0); **FTY**: 382 (71.4).	**Up to 36 months. Users on DMT at 36 months, n (%)** **DMF**: 307 (41.7%); **FTY**: 293 (54.8%).
**Zanghì et al.,** [37]	To evaluate the efficacy and safety profile of OCR, RTX, and CLA employed as NAT exit strategies in people RRMS at high risk for PML	Retrospective cohort study	Data from 11 tertiary Italian MS centres	CLA, OCR, (RTX)	**Users, n** **OCR**: 64; **RTX**: 36; **CLA**: 20.	**Mean (SD), years** **OCR**: 24.4 (9.5); **RTX**: 23.7 (9.9); **CLA**: 26.5 (10.2).	**Women, n (%)** **OCR**: 42 (65.6); **RTX**: 26 (72.2); **CLA**: 13 (65).	**Median [IQR], months** **OCR**: 18 [15–19]; **RTX**: 17 [14–20]; **CLA**: 16 [13–18].

AHSCT: autologous hematopoietic stem cell transplantations; ALE: alemtuzumab; AZA: azathioprine; CLA: cladribine; CYC: cyclosporine; DMF: dimethyl fumarate; DMT: disease-modifying treatment; FTY: fingolimod; GA: glatiramer acetate; IFN-beta: interferon-beta; IQR: interquartile range; MS: multiple sclerosis; MTX: mitoxantrone; NAT: natalizumab; OCR: ocrelizumab; pwMS: people with MS; RRMS: relapsing-remitting MS; RTX: rituximab; SAE: severe adverse event; SD: standard deviation. DMTs between brackets: exposure included in a study but not approved by the European Medicines Agency as DMT for MS

### Risk of bias in studies

**Fig. 2:**
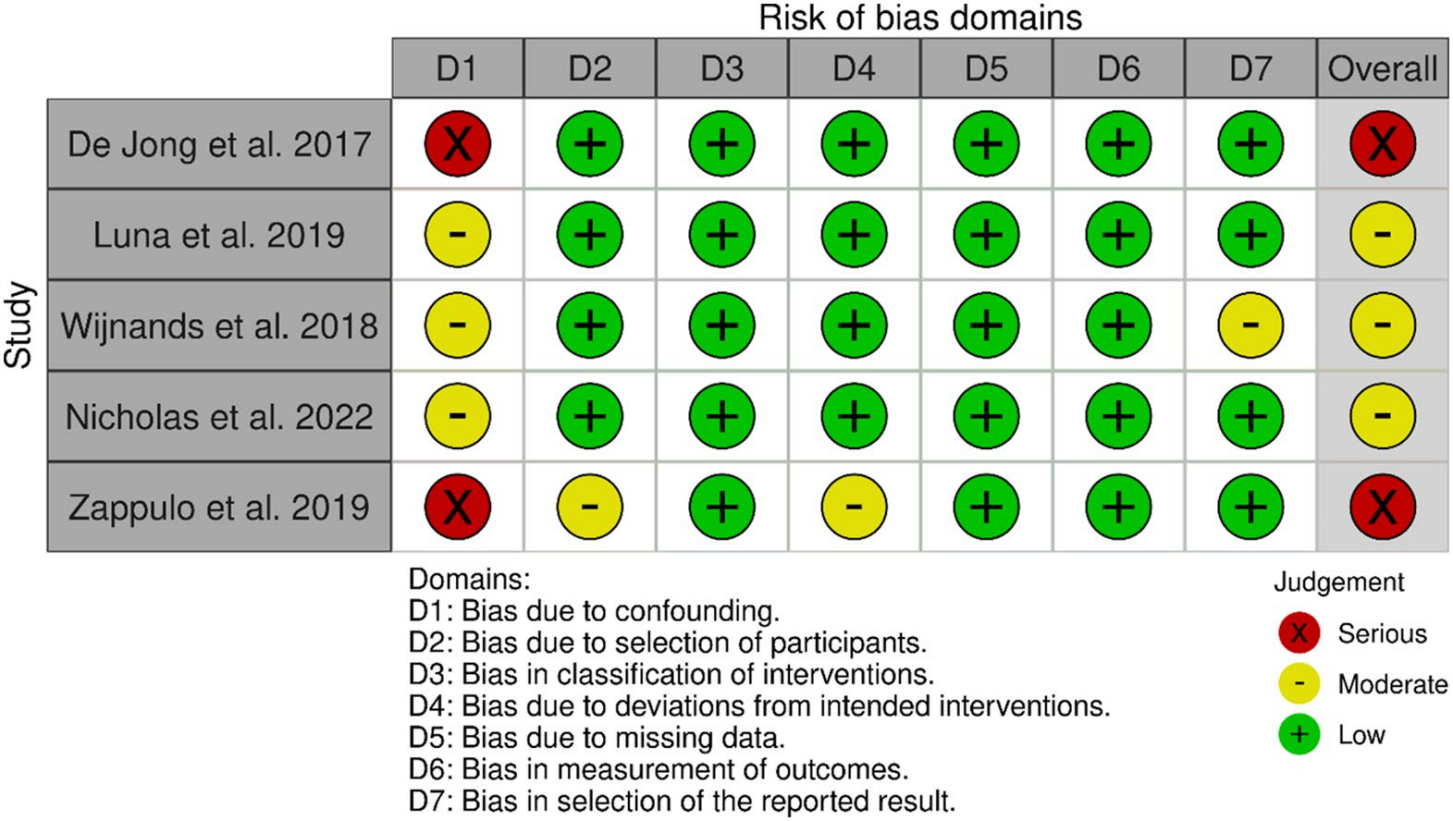
Risk of bias assessment for each included study with relative risk reported

Of the five studies with relative risk of infection on different DMTs, three were judged at moderate risk of bias and two at serious risk of bias (Fig. 2).

### Results of individual studies

#### Distribution of DMT contrasts

**Fig. 3:**
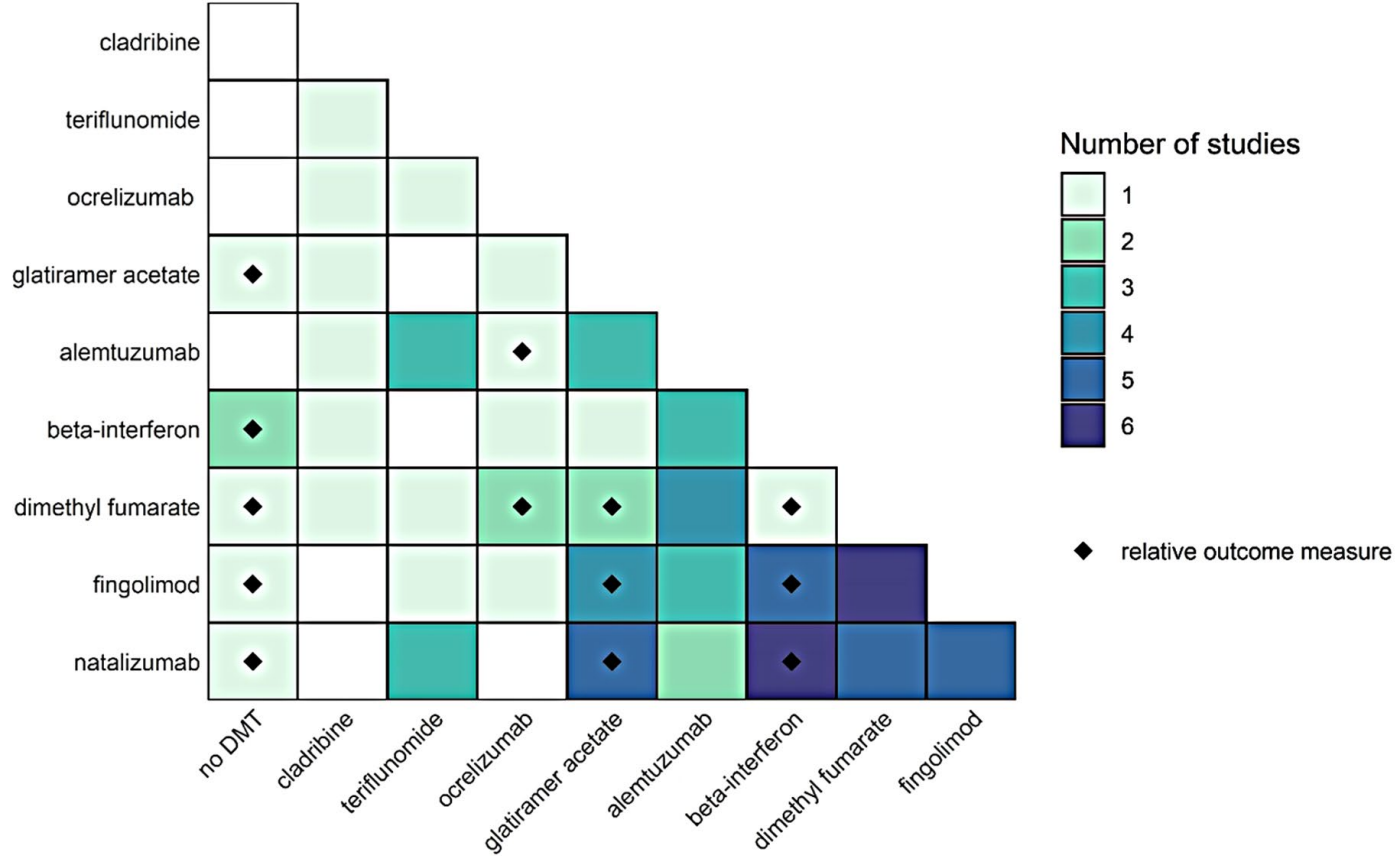
Distribution of disease-modifying treatment (DMT) contrasts over observational studies reporting on the occurrence of infections on DMT, by contrast in DMT exposure. A black diamond indicates that a relative risk measure was reported at least once. Studies reporting multiple DMT contrasts contributed to the respective multiple matrix cells. DMT exposure categories comprising multiple DMTs contributed to each contrast in the matrix that was possible through the component DMTs

For each contrast between DMT exposures, we noted how many observational studies reported the occurrence of infection and whether the results included a relative risk (Fig. 3). In total, 9 different DMTs were studied: alemtuzumab, beta-interferon, cladribine, dimethyl fumarate, fingolimod, glatiramer acetate, natalizumab, ocrelizumab, and teriflunomide. The distribution of the number of studies over the 45 possible contrasts was: 9 (20%) contrasts not studied, 19 (42.2%) contrasts once, 4 (8.9%) contrasts twice, 5 (11.1%) contrasts 3 times, 2 (4.4%) contrasts 4 times, 4 (8.9%) contrasts 5 times, and 2 (4.4%) contrasts 6 times. The most frequently contrasted DMTs overall were natalizumab (27 times over 12 studies) and fingolimod (26 times over 15 studies); the least frequently contrasted DMTs overall were cladribine (6 times over 3 studies) and ocrelizumab (8 times over 4 studies). However, a relative outcome measure of infection risk was reported most frequently for beta-interferon (14 times over 3 studies), and glatiramer acetate and natalizumab (each 12 times over 2 studies); and least frequently for cladribine and teriflunomide (each 0 times).

#### Distribution of types of infection

For each infection type, we noted how many observational studies assessed the occurrence on one DMT versus at least one other and whether the study was one of the five reporting a relative risk, or one of the 17 reporting the rate or number of events. The most assessed infection types were any (*n* = 14 studies), neurological (*n* = 11 studies, of which 10 reported on progressive multifocal leukoencephalopathy as outcome), and respiratory (*n* = 9 studies). The only specific infection type with reported relative risk was respiratory infection (1 study).

**Table 2:** Distribution of types of infection among observational studies assessing the occurrence of infection during DMT use for MS

Infection type	Studies^a^ with relative risks (*n* = 5)	Studies with rates or number of events (*n* = 17)
Any/unspecified	5	9
Neurological	0	11
Respiratory	**1**	**8**
Urinary	0	8
Genital	0	5
Skin and subcutaneous	0	4
Gastrointestinal	0	3
Herpes viral	0	8
Sepsis	0	1

^a^Studies assessing multiple infection types contributed to the respective multiple rows in the table

#### Infection risk on natalizumab: observational data versus network meta-analysis of trial findings

**Fig. 4:**
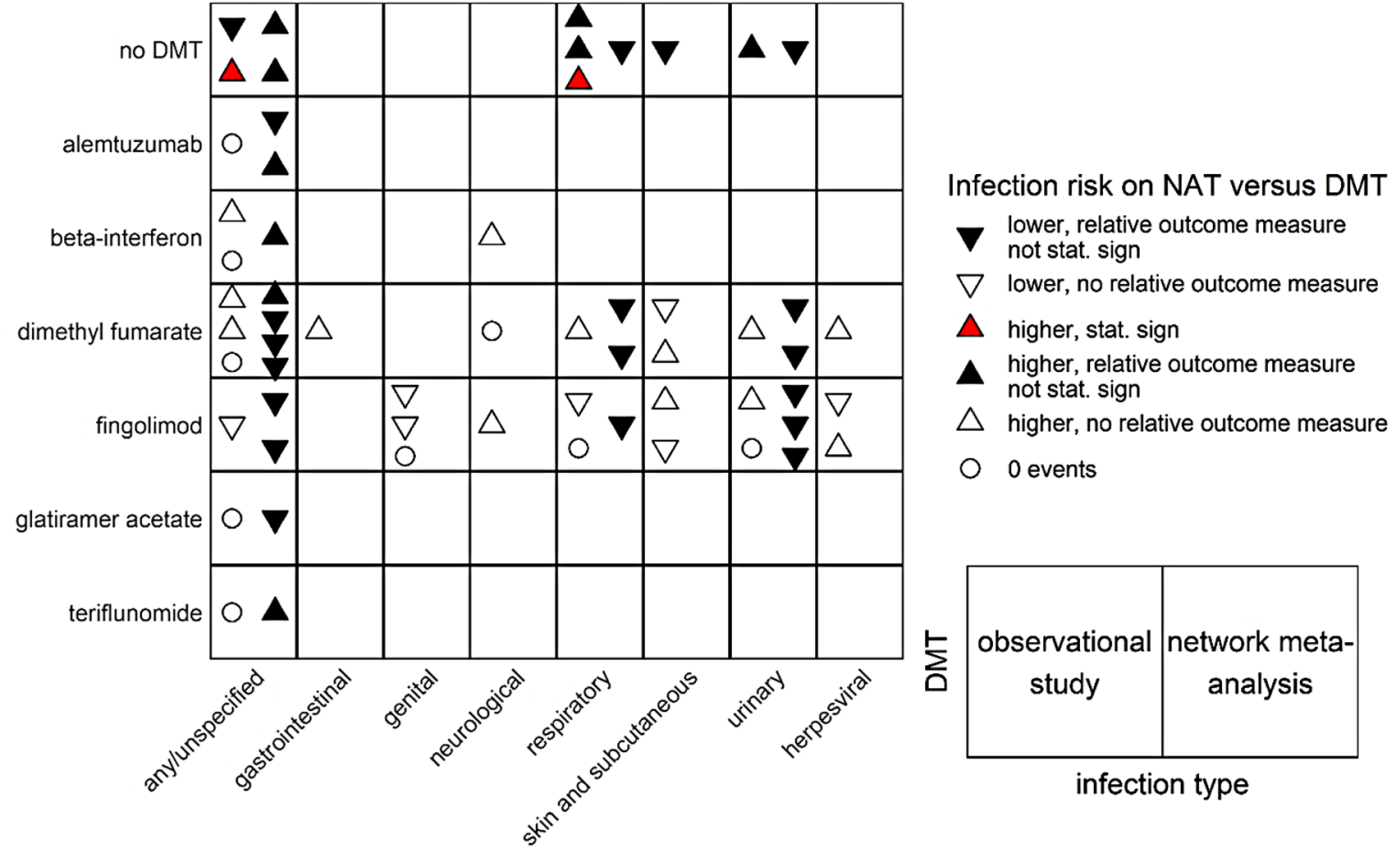
Infection risk on one of seven disease-modifying treatment (DMT) exposure categories compared to infection risk on natalizumab (NAT), from observational studies versus a recent network meta-analysis. If an observational study or the meta-analysis reported multiple outcomes or dosages belonging to a selected infection type or DMT, each result was included separately and represented by a triangle or circle

Natalizumab was the most studied DMT contrasting 7 other DMT exposures; observational data for natalizumab versus another DMT for 8 infection types were available for 23 of the 56 possible combinations (Fig. 4). For example, for the comparison between natalizumab and fingolimod on their risk of urinary infections, three estimates were available from the trial meta-analysis [5] (all showing a lower, non-statistically significant risk of natalizumab) while there were two observational studies found (one showing a higher, non-statistically significant risk of natalizumab and one with zero events). Observational findings on any, respiratory, and urinary infection could be compared to trial data for 13 combinations: associations were 3 times in similar direction, 5 times in opposite direction, one time observational data were contradictory, one time trial data were contradictory and 3 times there were 0 events in the observational study (Fig. 4). Infection definitions and severity from the same infection type differed between the studies, e.g. respiratory infections included upper respiratory infection, bronchitis and bronchiolitis, pneumonia, and nasopharyngitis.

## Discussion

Our review showed that observational study data on the risk of infection in pwMS on DMT are sparse, limited in infection types and heterogeneous in infection definition. We found that only 5/22 (22.7%) of the observational studies assessing the occurrence of infection with at least one contrast in DMT exposure included a relative risk; the others only provided counts. The latter studies should be considered as providing a lower level of evidence, because they were not set up to investigate comparative safety using standard epidemiological tools such as confounding adjustment. For the five that were aimed to provide a relative risk, our risk of bias assessment showed three at moderate risk of bias and two at serious risk. The domain with the highest risk of bias was bias due to confounding, which determined the overall risk of bias for each of the five studies. We found that 9/45 (20%) possible DMT contrasts were not studied at all, and 19/45 (42.2%) were included in only one observational study. It was thus not possible to conduct a meta-analysis of the relative risk of infection on DMTs for MS using observational data. In contrast, a meta-analysis has been conducted using trial data and with newer DMTs that were not in the observational studies included in this review (e.g. ofatumumab, ozanimod, and ponesimod) [5].

Most observational studies only investigated a combined outcome measure of any infection type. This is not ideal because infection management is specific to the infection type. Respiratory infection was the only specified infection type for which the relative risk was assessed, in one study. Neurological infection was the most frequently specified infection type, which is likely due to the well-studied risk of progressive multifocal leukoencephalopathy on natalizumab [38]. The absence of data on the occurrence of specific infections is partly inherent to the observational nature of the studies: newer DMTs cannot be studied until the amount of follow-up time is sufficient and therefore sample sizes are often limited. Also, DMT effectiveness is of great research interest [39], while it seems that less observational research has been conducted on infection as part of the safety as well as effectiveness of DMTs: better control of underlying MS disease processes may be reflected in lower rates of infections resulting from organ dysfunction. Given that infections can worsen the MS disease course [40], they are an important safety aspect worthy of further study as a broader and more general concern than that triggered by one specific infection due to one specific DMT, as was the case for progressive multifocal leukoencephalopathy on natalizumab. UTI in particular deserves more attention given its high disease burden. This burden is evident from the high UTI prevalence at the first MS diagnosis and after, the increase in risk of UTI risk due to the urinary incontinence that can occur over the MS disease course, and the increased risk of UTI-related hospitalization and death among pwMS [40]. Moreover, bladder dysfunction has been identified as a patient-relevant outcome because of its potentially disabling consequences [41].

For the most studied DMT, natalizumab, observational data could be compared to trial data for 13/56 (23.2%) possible combinations of risk of one of eight infection types on natalizumab versus one of seven other DMT exposures. The associations were in similar direction in three combinations. Point estimates of the relative risks (compared to no DMT) for respiratory and urinary tract infections were in opposite direction compared to trial data. Differences in the point estimate of the risk of infections between observational studies and trials may result from differences in outcome definition, but also greater heterogeneity in the study population of observational studies. Trial participants are likely more homogeneous than real-world users in age, disease activity, and prior DMT use [42]. This is relevant because (1) it has been hypothesized that successive use of different DMTs may increase the risk of infection [43]; (2) ageing increases the risk of infection [44]; and (3) organ function can decrease over the course of MS in absence of relapse [45], which increases the risk of infection [44]. As an example: we found that natalizumab compared to no DMT increased the risk of respiratory infection in observational research, but not based on trial data [5]. In the observational study involved [19], the outcome ‘respiratory infection’ was covered in three specific definitions: upper respiratory tract infection, bronchitis and bronchiolitis, and pneumonia. The point estimate of the hazard ratio (adjusted for sex, age, socioeconomic status at first demyelinating event (index date), and number of comorbidities) of each outcome for natalizumab versus no DMT was greater than 1 for all three specific outcomes, with statistical significance in the case of upper respiratory infection. The meta-analysis of trial data included only nasopharyngitis as outcome of respiratory infection and reported the opposite direction of effect (although not statistically significant) for the odds of natalizumab versus no DMT [5]. In the observational study, follow-up time was up to 17.7 years from index date, the mean age at index date of people who contributed natalizumab-exposed follow-up time (*n* = 5077 people) was 34.5 years and 47.8 years among people who contributed non-DMT exposed time (*n* = 100 people). The meta-analysis included one 2-year trial of natalizumab (versus placebo) [46], in which the study population comprised only people with relapsing MS (at least one relapse within the year prior to study start), the mean age was 35.6 years in the natalizumab arm (*n* = 627 people) and 36.7 years in the placebo arm (*n* = 315 people), median disease duration was 5.0 years in the natalizumab arm and 6.0 years in the placebo arm, and people had been excluded if treated with cyclophosphamide or mitoxantrone in the previous year; interferon beta, glatiramer acetate, cyclosporine, azathioprine, methotrexate, or intravenous immune globulin in the previous 6 months; or with interferon beta or glatiramer acetate for more than 6 months. The discrepancy in findings on the risk of respiratory infection on natalizumab versus no DMT could thus be due to the difference in outcome definitions between the observational study and the trial; a difference in short- (trial) and long-term effects (observational study); or a difference in effect on people with more active disease (trial), higher age (observational study), or prior DMT use (observational study).

A key strength of this study is the comprehensive approach to include observational studies of the risk of infection among pwMS. The risk of bias assessment of the studies with relative risk provides context for the strength of the evidence, and the additional inclusion of studies reporting counts of infections adds depth to the picture. One limitation of this study is that the small number of observational studies limited the reliability of the overall risk of infection on DMT (Fig. 4). Moreover, the available real-world data were collected in relatively large part from natalizumab users. This may limit the generalizability of the findings to the whole population with MS, as natalizumab may be considered for pwMS with certain characteristics (e.g. DMT-naïve, with suboptimal treatment response to another DMT, or at risk of aggressive MS disease course) and not others (e.g. those at higher age or with progressive MS) [47]. Another limitation is that it was not possible to investigate possible correlations between duration of MS, DMT exposure, or follow-up; and occurrence of opportunistic infection or severity of infection. Lastly, we may have missed studies that did report the occurrence of infection on DMT for MS, but for which this was not explicitly mentioned in the title, abstract, or keywords. This is also true for studies written in a language other than English.

Real-world data from MS registries and cohorts can help inform the benefit-risk ratio that plays a crucial role in the personalized DMT decision. The establishment of MS registries may resolve issues around the fragmented nature of other real-world data sources, which often need to be combined in order to gather the necessary data for a well-designed observational pharmacoepidemiologic study of exposure and infection. Observational studies are well-suited to fill the evidence gap regarding the infection risk of DMTs because they can facilitate a bigger, broader study population and longer follow-up duration than trials, thus yield bigger sample size and power [39]. Observational research may thus be better suited than trials for the study of long-term effects of DMT exposure on risk of frequently occurring, mild infections such as UTI; but also of rare, severe infections such as progressive multifocal leukoencephalopathy, which has emerged as a significant risk of DMT use in the treatment of MS. Beyond these direct advantages of observational studies over trials, observational data allow for understanding of DMT switching patterns [39]. In turn, this could improve understanding of how the infection risk is affected by DMT history and person characteristics underlying the treatment strategy. Moreover, the use of observational data for studies of heterogeneous treatment effects of DMT– a current research topic in light of personalized treatment for MS– yields higher generalizability than trial data [39]. Developments in observational data for MS facilitate future research. First, statistical methods are improved to achieve balance in person characteristics at and after baseline through MS-specific investigation of the modelling of a propensity score and its applications. Second, the use of a standardized template for real-world data on MS is being investigated [48], which will be especially helpful in data access to accurate information regarding DMT initiation and discontinuation. Third, multiregional and international databases are being created for real-world data on MS, adding to the generalizability of real-world studies in MS [39].

In this systematic review, we found that the relative risk of infection in pwMS on DMT has not been studied frequently using observational data. Evidence on newer DMTs and specific infection types is particularly limited. The growing availability of real-world data on MS and DMT use provides an opportunity to study this relevant safety outcome of DMTs, especially in populations underrepresented in clinical trials.
